# A case report of youth-onset lipoprotein glomerulopathy with *APOE* Chicago mutation

**DOI:** 10.1186/s12882-024-03515-z

**Published:** 2024-03-06

**Authors:** Yuhao Liu, Yaqi Cheng, Yubing Wen, Chao Li, Gang Chen, Mingxi Li

**Affiliations:** Department of Nephrology, State Key Laboratory of Complex Severe and Rare Diseases, Peking Union Medical College Hospital, Peking Union Medical College and Chinese Academy of Medical Sciences, Beijing, 100730 China

**Keywords:** Lipoprotein glomerulopathy, *APOE* gene, *APOE* Chicago mutation

## Abstract

**Background:**

This article reports an extremely rare case of lipoprotein glomerulopathy (LPG) with apolipoprotein E gene (*APOE*) Chicago mutation in a young Chinese male. Only five cases or families with *APOE* Chicago mutations have been reported in the literature.

**Case presentation:**

The young male patient is manifested with nephrotic syndrome, accompanied by hyperlipidemia with a preferable increase in triglycerides and elevated ApoE level. Renal biopsy of the patient showed highly dilated glomerular capillaries filled with vacuolar lipids, segmentally fused podocyte foot processes, vacuolar degeneration of renal tubular epithelial cells and absence of electron-dense material, which indicates the diagnosis of LPG. Whole-exome gene sequencing identified the heterozygous mutation of NM_000041.4:c.494G > C (p.Arg165Pro), which is in the exon 4 of the *APOE* gene and also known as *APOE* Chicago mutation, a rare mutation of LPG. Further family pedigree gene analysis clarified that the mutation was inherited from the patient’s mother, who does not have high ApoE levels or renal manifestations. This is also consistent with the incomplete penetrance of *APOE* gene mutations in LPG. Under lipid-lowering treatments, including a low-fat diet and fenofibrate, the patient’s urinary protein was partially controlled, and the albumin level was recovered.

**Conclusion:**

Patients with nephrotic syndrome and elevated ApoE levels should be prompted into renal biopsy to avoid delay of appropriate treatment and unnecessary use of glucocorticoids. This case of LPG was diagnosed by renal biopsy and further verified with genetic sequencing. The timely diagnosis and treatment improved the patient’s symptoms. This case is one of only six reported LPG cases or families with *APOE* Chicago mutation in the world.

## Background

Lipoprotein glomerulopathy (LPG) is an inherited glomerular disease caused by mutations in the *APOE* gene, usually with clinical manifestations of dysregulated lipid metabolism, proteinuria, and renal insufficiency. Diagnosis of LPG relies primarily on renal pathology, with typical lipoprotein thrombosis within dilated glomerular capillary loops. The first 2 cases of LPG were reported by Japanese scholar Saito et al. in the same family in 1989 [1]. The patients were manifested with proteinuria and edema, and their renal histology was characterized by marked dilatation of capillary lumina occupied with lipid droplets. Due to the iconic glomerular lipoprotein thrombi, Saito et al. named the disease lipoprotein glomerulopathy.

Oikawa et al. later discovered the first LPG pathogenic gene in 1997, which was a missense mutation in the exon 4 of *APOE*, causing the 145th amino acid arginine in the mature ApoE protein to be replaced by proline. This mutation was named *APOE* Sendai (Arg145Pro) [2]. With a deepened understanding of LPG and widespread application of gene sequencing, 17 *APOE* gene mutations have been related to LPG and more than 200 cases have been reported worldwide up to date. LPG epidemiology is mostly based on case reports, of which are more concentrated in East Asia. The estimated prevalence of LPG in Japan is approximately 3.74 per 10 million people and in China is 1.43 per 10 million people [3]. Among them, Chinese LPG patients have the highest incidence of APOE Kyoto (Arg25Cys) mutations, while Japanese LPG patients mainly have APOE Sendai (Arg145Pro) mutations [3, 4].

## Case presentation

The young Chinese male patient developed facial and lower limb edema at the age of 26, accompanied by loss of appetite, increased foam in the urine, and rapid gain of body weight. No gross hematuria nor dyspnea were evident. The blood routine test at his local hospital was normal. Urine routine: protein 3+, occult blood 2+; 24-hour urine protein (24hUP): 3.84 g. Blood biochemistry: albumin (Alb) 29.9 g/L, creatinine (Cr) 83µmol/L (57–97 µmol/L), total cholesterol (TC) 6.83mmol/L, triglyceride (TG) 6.37mmol/L. No xanthoma was present despite his hyperlipidemia. Nephrotic syndrome was diagnosed, and screening for hepatitis B, antinuclear antibody profile, and anti-phospholipase A2 receptor antibody were all negative. After treatment with albumin supplementation and diuretics, his edema was significantly alleviated, whereas the foam in the urine persisted. Reluctant to renal biopsy, the patient was instructed to follow a low-fat low-cholesterol diet and prescribed irbesartan to alleviate both proteinuria and hypertension, which was accidentally discovered at the age of 23. However, the patient did not strictly follow the treatment and seldom monitored his blood pressure. During irregular follow-up, the patient experienced a relapse of edema and exacerbation of foamy urine. Lab tests showed Alb 31.3/L, Cr 93µmol/L, and 24hUP 7.24 g. Apolipoprotein E (ApoE) level was found to be increased as 5.97 mg/dL (2.7–4.9 mg/dL), whilst Apolipoprotein A1 and Apolipoprotein B levels were within the normal range.

**Fig. 1:**
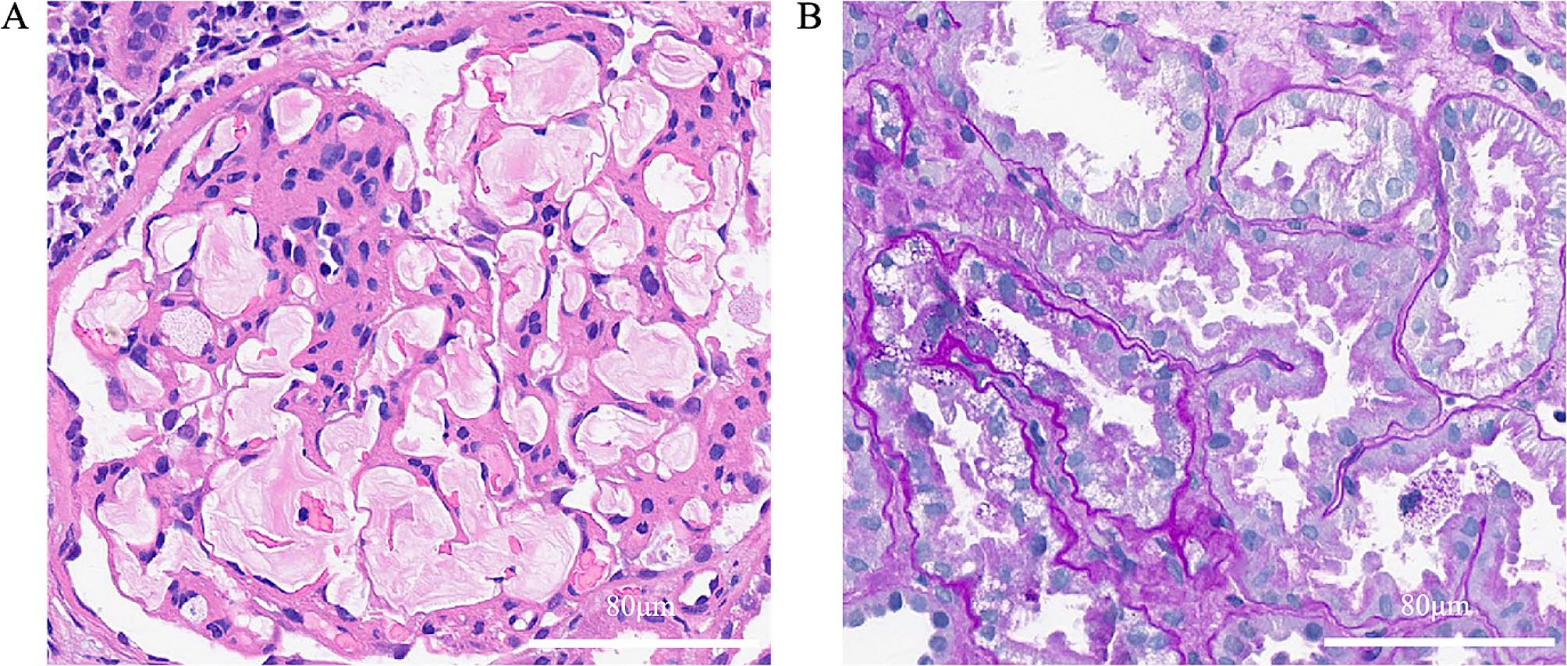
The patient’s renal pathology in light microscopy. (**A**) laminar lipoprotein emboli in dilated glomerular capillaries shown in H&E staining. (**B**) vacuolar degeneration of renal tubular epithelial cells, renal tubule lumen expansions and disappearance of bristle margins shown in PAS staining. H&E staining: Hematoxylin Eosin staining, PAS staining: Periodic Acid-Schiff staining

**Fig. 2:**
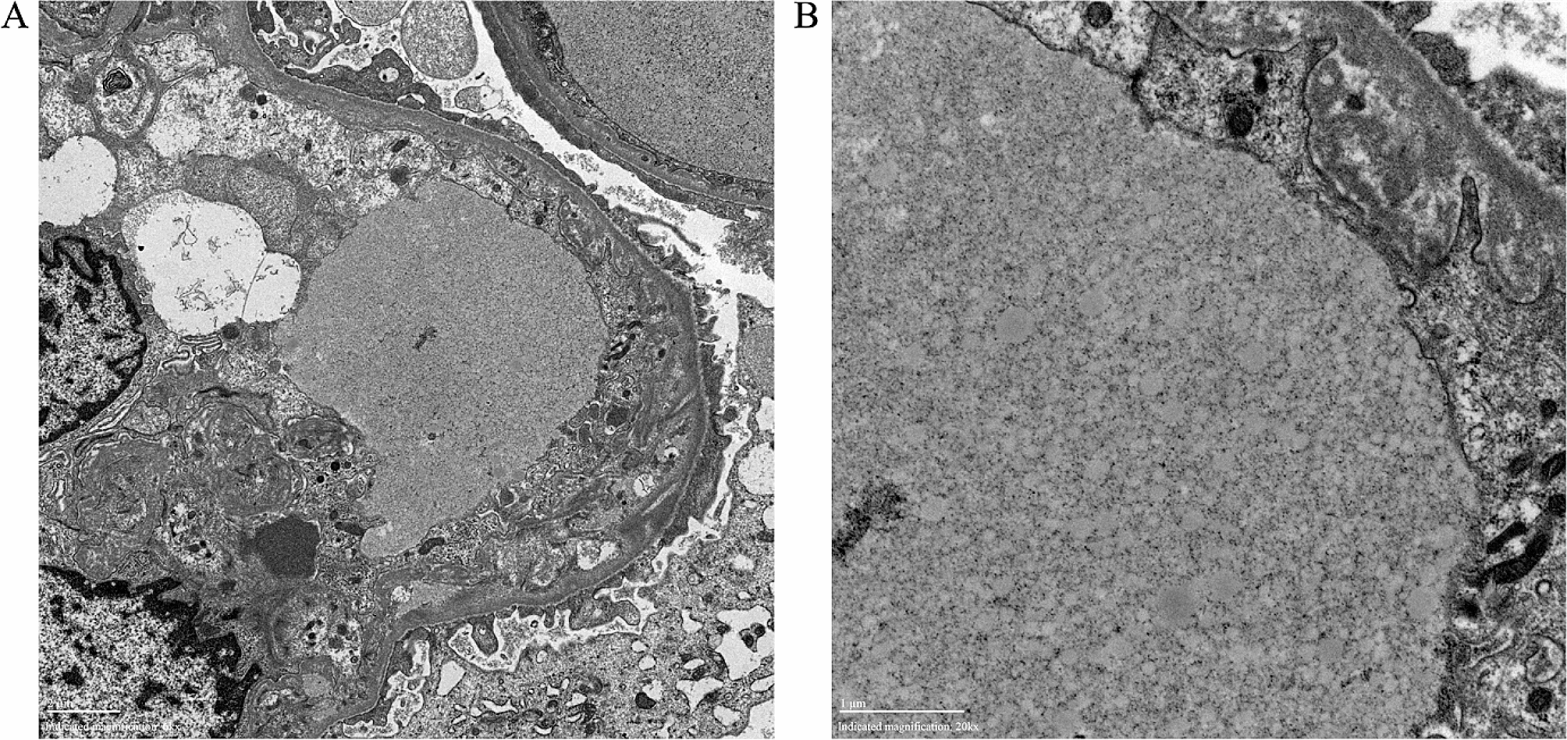
The patient’s renal pathology in electron microscopy

Due to further increase of urine protein, renal biopsy was conducted and showed segmental sclerosis in 3 out of 67 glomeruli in the light microscopy. The patient’s glomeruli were enlarged, and glomerular capillaries were highly dilated filled with vacuolar or laminar lipids. Endothelial cells were swollen with vacuolar degeneration, and glomerular mesangial cells and matrix exhibited moderate to severe hyperplasia with no eosinophilic deposit. Renal tubular epithelial cells showed vacuolar degeneration with some renal tubule lumen expansions, disappearance of bristle margins, and focal atrophy. The interstitium fibrosis was mild to moderate with focal inflammatory cell infiltration. The walls of renal arterioles are slightly thickened (Fig. 1). Immunofluorescence: IgM (+/-), lgG, IgA, C3, C1q, Fib, Alb (-). Electron microscopy: dilated glomerular capillary loop lumen was filled with large amounts of lipid vacuoles, podocyte foot process was segmentally fused, and no electron dense material was found (Fig. 2).

(A) 6000X, lipoprotein emboli in the glomerular capillary lumen, swollen endothelial cells, expanded inner loose layer, and segmentally fused foot process. (B) 20000X, lipoprotein emboli in the capillary lumen contain a large number of lipid vacuoles.

Considering the increased ApoE level and classic renal pathology, the diagnosis of LPG is of high likelihood. Thus, the patient was treated with fenofibrate in addition to irbesartan. Subsequent follow up revealed partial alleviation with Alb 45 g/L, Cr 77µmol/L, 24hUP 3.09 g, TC 4.12mmol/L and TG 2.06mmol/L, further support the LPG diagnosis.

LPG is caused by genetic mutations of APOE. But the mutations usually do not include the major APOE mutation E2 based on the amino acid residue at site 158. Accordingly, genotype E3/E3 or E3/E4 are identified in LPG as in our patient. Therefore, whole exon sequncing was applied and revealed a heterozygous mutation of the patient in *APOE* gene NM_000041.4:c.494G > C(p.Arg165Pro). According to the guidelines of the American College of Medical Genetics and Genomics (ACMG), this variant is classified as a suspected pathogenic variant (PS4_Supporting + PM1 + PM2 + PP4). Owing to the absence of family renal disease history, exon sequencing was also performed on the patient’s parents. The patient’s father has wild-type *APOE*, while the patient’s mother carries the same heterozygous mutation in NM_000041.4:c.494G > C(p.Arg165Pro), clarifying the inheritance of the pathogenic mutation.

Nevertheless, unlike the patient, his mother exhibited normal ApoE levels and preserved kidney function with no proteinuria and hematuria. According to the Online Mendelian Inheritance in Man database, some individuals with heterozygous LPG-pathogenic *APOE* mutation may not develop LPG, which is incomplete penetrance and in line with the genotype and clinical manifestation mismatch in the patient’s mother.

## Discussion and conclusion

Here we report a young male Chinese LPG case with clinical manifestation of nephrotic syndrome and hyperlipidemia with especially elevated TG and ApoE levels. The diagnosis of LPG was based on the renal biopsy showing typical lamellated lipoprotein thrombi in his glomerular capillary lumina. Further whole exon sequencing of the patient identified the pathogenic *APOE* Chicago (Arg147Pro) mutation, which has only been reported in five patients/families so far and their characteristics are summarized in Table 1.

**Table 1:** Characteristics of previous cases with *APOE* Chicago mutation

	Onset age/Sex/Ethnicity	*APOE* mutations	Clinical data	Family history	Ref
Case 1	33/Male/Japanese	*APOE* Chicago, *APOE*2/ *APOE*(Glu3Lys)	24hUP 3-6 g, Alb 30 g/L, eGFR 62.7 ml/(min·1.73m^2^); kidney transplant at 36	No	[5, 6]
Case 2	32/Male/Mexican	*APOE* Chicago	24hUP 9.7 g, Alb 29.1 g/L, eGFR 57 ml/(min·1.73m^2^)	Family history of premature renal failure without gene information	[7]
Case 3	51/Female/Japanese	*APOE* Chicago, APOE (Glu3Lys)	Urine albumin/creatinine ratio 4120 mg/g Cr, Alb 36 g/L, eGFR 90 ml/(min·1.73m^2^)	Patient’s mother with same mutation but no clinical manifestation.	[8]
Case 4	50/Male/Chinese	*APOE* Chicago	24hUP 5.7 g, Alb 31.2 g/L, eGFR 51.9 ml/(min·1.73m^2^)	No	[9]
Case 5	7/Male/Chinese	*APOE* Chicago	24hUP 2.5 g, Alb 22.3 g/L, eGFR N/A, Cr 48.5µmol/L	Paternal gene mutation; family history of proteinuria and renal failure.	[10]

*APOE* apolipoprotein E, 24hUP: 24-hour urine protein, Alb: albumin, Cr: creatinine, eGFR: estimated glomerular filtration rate.

The *APOE* gene of humans is located on chromosome 19 and comprised of 4 exons, encoding an N-terminal signal peptide containing 18 amino acids and a mature ApoE protein consisting of 299 amino acids. Therefore, the *APOE* Chicago mutation (p. Arg165Pro) is often expressed as Arg147Pro in the mature protein. Amino acids 140–150 in the N-terminal domain are low-density lipoprotein (LDL) receptor binding sites, and amino acids 244–272 in the C-terminal domain are binding sites for lipids [11]. As a vital component of various lipoproteins (chylomicrons, very low-density lipoproteins, and medium-density lipoproteins), ApoE binds to LDL receptors or LDL receptor-related proteins and mediates the uptake of triglyceride-rich lipoproteins by hepatocytes from circulation. As a result, ApoE dysfunction causes elevation of blood lipids.

Many LPG-pathogenic *APOE* gene mutations are in or near its binding site of LDL receptor, such as *APOE* Sendai (Arg145Pro), *APOE* Chicago (Arg147Pro) and *APOE* Las Vegas (Ala152Lys). These mutations mitigate the binding ability of ApoE protein to LDL receptor, resulting in elevations of ApoE in the circulation, and could be an essential mechanism in the LPG development [12]. However, despite the worldwide prevalence of hyperlipidemia, LPG is an extremely rare disease, suggesting that the sole increase of ApoE-containing lipoprotein is not adequate for LPG pathogenesis.

Mutated ApoE2 (Arg158Cys) exhibits only 2% affinity to LDL receptor compared to the wide type ApoE3 isoform [13], and more than 95% of type III hyperlipoproteinemia individuals carry homozygous *APOE2* mutation [14]. However, most of homozygous *APOE2* carrier are normolipidemic and less than 10% of them show type III hyperlipoproteinemia [14, 15]. Additionally, several cases are reported to have glomerular lesions as called ApoE2 homozygote glomerulopathy, which is characterized by foam cell infiltration and different from LPG [16]. These facts further illustrate the complex pathogenesis of LPG.

Physiological ApoE protein is highly helical and can transform between variable tertiary structures to achieve various physiological functions. Studies have found that many LPG pathogenic *APOE* mutations, such as *APOE Kyoto* (Arg25Cys), *APOE* Sendai (Arg145Pro), and *APOE* Chicago (Arg147Pro), significantly reduce the helicity of ApoE protein and increase its thermodynamic instability [17, 18]. Mutated ApoE is more prone to denaturation at physiological temperature and possesses significantly higher aggregation tendency [17, 18]. Moreover, mutated ApoE also exhibits increased affinity to endothelial cells, as found in *APOE* Kyoto (Arg25Cys) and *APOE* Chicago (Arg147Pro), which help drive lipoproteins to deposit primarily in the capillary lumina of glomerulus and eventually cause LPG [7, 19].

In summary, LPG-pathogenic *APOE* mutations interfere with hepatocyte uptake of mutated ApoE-containing lipoproteins, resulting in increased levels of ApoE in circulation. These mutated lipoproteins are with higher aggregation tendency and prone to interact with glomerular capillary endothelial cells and deposit in the glomerulus, which together lead to the pathogenesis of LPG.

LPG is an extremely rare disease with most cases in Eastern Asia. LPG treatment aims to reduce proteinuria and delay the progression of renal dysfunction. Glucocorticoids and immunosuppressants are not recommended while lipid-lowering treatment has been shown to alleviate proteinuria and even reverses renal capillary lipoprotein thrombi in LPG [20]. Additional treatments include renin-angiotensin-aldosterone system inhibitors and plasma adsorption/exchange therapy have also been proven to be effective in LPG monitoring [21, 22].

Through diagnosis and treatment of this LPG case, it is suggested that patients with unexplained nephrotic syndrome and elevated ApoE levels should receive renal biopsy actively to avoid unnecessary immunosuppressive treatments. Studies are needed to specify influences of various *APOE* mutations in LPG and functional verification are guaranteed to further improve our understanding of this rare disease.

## Data Availability

The datasets used and/or analyzed during the current study available from the corresponding author on reasonable request.

## Abbreviations

ApoE: Apolipoprotein E
LPG: Lipoprotein glomerulopathy
24hUP: 24-hour urine protein
Alb: Albumin
Cr: Creatinine
TC: Total cholesterol
TG: Triglyceride
